# mRNA SARS-CoV-2 Vaccination Before vs During Pregnancy and Omicron Infection Among Infants

**DOI:** 10.1001/jamanetworkopen.2023.42475

**Published:** 2023-11-10

**Authors:** Orlanda Goh, Deanette Pang, Janice Tan, David Lye, Chia Yin Chong, Benjamin Ong, Kelvin Bryan Tan, Chee Fu Yung

**Affiliations:** 1 Department of Internal Medicine, Singapore General Hospital, Singapore; 2 SingHealth Duke-NUS Medicine Academic Clinical Programme, Singapore; 3 SingHealth Duke-NUS Global Health Institute, Singapore; 4 Ministry of Health, Singapore; 5 Lee Kong Chian School of Medicine, Nanyang Technology University, Singapore; 6 Department of Infectious Diseases, Tan Tock Seng Hospital, Singapore; 7 Yong Loo Lin School of Medicine, National University of Singapore, Singapore; 8 National Centre for Infectious Disease, Singapore; 9 Infectious Disease Service, Department of Paediatrics, KK Women’s and Children’s Hospital, Singapore; 10 SingHealth Duke-NUS Paediatrics Academic Clinical Programme, Singapore; 11 Duke-NUS Medical School, Singapore; 12 Saw Swee Hock School of Public Health, National University of Singapore, Singapore

## Abstract

**Importance:**

Infants younger than 6 months are at risk of severe SARS-CoV-2 infection. Data are lacking on the optimum timing for maternal vaccination and estimated effectiveness against Omicron variants, including XBB, for infants.

**Objective:**

To investigate maternal vaccination against Omicron variants, including XBB, and the association of vaccination timing during pregnancy vs prior to pregnancy and risks of SARS-CoV-2 infection among infants aged 6 months or younger.

**Design, Setting, and Participants:**

This population-based cohort study was conducted between January 1, 2022, and March 31, 2023. Singapore’s national dataset was used to study infants born at greater than 32 weeks’ gestation between January 1, 2022, and September 30, 2022. The study included infants whose parents had a confirmed SARS-CoV-2 infection from the date of birth up to 6 months of age. Of 21 609 infants born during this period, 7292 (33.7%) had at least 1 parent infected with SARS-CoV-2 before the age of 7 months. Statistical analysis was performed from April to July 2023.

**Exposure:**

Infants’ mothers were unvaccinated, vaccinated prior to pregnancy, or vaccinated with a messenger RNA (mRNA) SARS-CoV-2 vaccine during pregnancy.

**Main Outcome and Measure:**

Infants were considered infected if they had a positive polymerase chain reaction test.

**Results:**

Among 7292 infants included in this study, 4522 (62.0%) had mothers who were Chinese, 527 (7.2%) had mothers who were Indian, 2007 (27.5%) had mothers who were Malay, and 236 (3.2%) had mothers who were other ethnicity; 6809 infants (93.4%) were born at full term, and 1272 infants (17.4%) were infected during the study period. There were 7120 infants (97.6%) born to mothers who had been fully vaccinated or boosted as of 14 days prior to delivery. The crude incidence rate was 174.3 per 100 000 person-days among infants born to mothers who were unvaccinated, 122.2 per 100 000 person-days among infants born to mothers who were vaccinated before pregnancy, and 128.5 per 100 000 person-days among infants born to mothers who were vaccinated during pregnancy. The estimated vaccine effectiveness (VE) was 41.5% (95% CI, 22.8% to 55.7%) among infants born to mothers vaccinated during pregnancy. Infants of mothers who received vaccination prior to pregnancy did not have a lower risk for infection (estimated VE, 15.4% [95% CI, −17.6% to 39.1%]). A lower risk for Omicron XBB infection was only observed among mothers vaccinated with the third (booster) dose antenatally (estimated VE, 76.7% [95% CI, 12.8% to 93.8%]).

**Conclusions and Relevance:**

In this population-based cohort study, maternal mRNA vaccination was associated with a lower risk of Omicron SARS-CoV-2 infection among infants up to 6 months of age only if the vaccine was given during the antenatal period. These findings suggest that mRNA vaccination during pregnancy may be needed for lower risk of SARS-CoV-2 infection among newborns.

## Key Points

**Question:**

Is maternal vaccination associated with a lower risk of infection with Omicron SARS-CoV-2 variants, including XBB, among infants up to 6 months of age?

**Findings:**

In this national population-based cohort study of 7292 infants aged 6 months or younger in Singapore, the estimated vaccine effectiveness in infants against Omicron SARS-CoV-2 variants, including XBB, from maternal messenger RNA (mRNA) SARS-COV-2 vaccination was 42%. A lower risk of infection was only found in infants when the vaccine was administered during pregnancy.

**Meaning:**

These findings suggest that mRNA vaccination during pregnancy may be needed to reduce the risk of SARS-CoV-2 infection among newborns.

## Teaser

This cohort study investigates the association of maternal vaccination against SARS-CoV-2 Omicron variants before vs during pregnancy with risk of infection among infants.

## Introduction

Infants aged 6 months or younger are at high risk of severe SARS-CoV-2 infection.^1,2^ In the United States, they accounted for 44% of all infant and children SARS-CoV-2 hospitalizations during Omicron dominant waves beginning in December 2021.^3^ Furthermore, the majority of the hospitalized infants aged 6 months or younger had no underlying comorbidity. Population-level vaccination has been extended to infants and children aged 6 months to 17 years with reported evidence of effectiveness.^4,5,6^ To date, SARS-CoV-2 vaccines for infants up to 6 months of age remain unavailable.

Maternal SARS-CoV-2 vaccination has been shown to prevent severe SARS-CoV-2 disease in pregnancy and reduce the risk of poor pregnancy outcomes.^7,8^ Aside from adhering to social distancing measures, it may be the only means of protection for infants through the transfer of maternal antibodies in cord blood.^9,10^ Higher antibody levels against SARS-CoV-2 have been found in infants whose mothers who were vaccinated during pregnancy.^11,12^ While studies have shown maternal vaccination is associated with lower infant SARS-CoV-2 infection and hospitalization, a better understanding of factors associated with lower risk is needed.^13,14^ Data on the optimum timing for maternal vaccination for infant protection and evidence on effectiveness against the Omicron XBB variant are lacking.^15^ Studies to date have also focused on receipt of vaccination during pregnancy while there is little information about infants whose mothers were fully vaccinated (including third doses) prior to pregnancy. Understanding this is critical for informing maternal SARS-CoV-2 vaccination policy specifically on whether vaccination is required for every pregnancy, especially in light of global vaccination hesitancy.^16,17^

We aimed to determine whether maternal vaccination with a messenger RNA (mRNA) vaccine was associated with a lower risk of Omicron variants, including XBB, among infants up to the age of 6 months. In addition, we investigated the association of vaccination timing during pregnancy vs prior to pregnancy and the risk of getting SARS-CoV-2 infection among newborn infants.

## Methods

### Study Population and Data Sources

**Figure 1.**
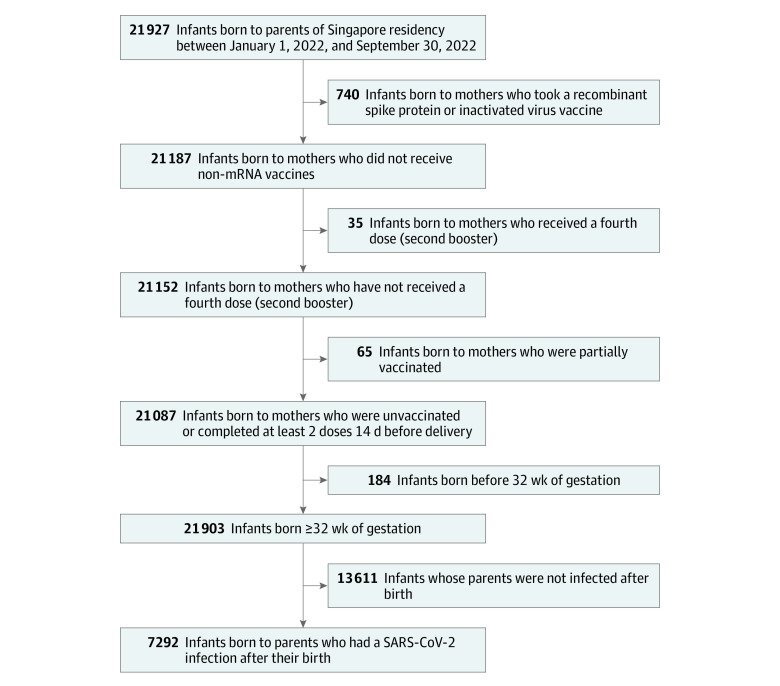
Inclusion and Exclusion Criteria mRNA indicates messenger RNA.

We conducted a retrospective cohort study between January 1, 2022, and March 31, 2023 (a period dominated by Omicron variants^18^) based on all infants born to registered Singapore citizens and permanent residents between January 1, 2022, and September 30, 2022. We excluded infants born to mothers who had incomplete demographics information, infants born to mothers who received non-mRNA vaccines, or completed only 1 vaccine dose or completed a second dose less than 14 days prior to delivery, and infants born before 32 weeks of gestation. To isolate the estimated effect of in-utero vaccination and minimize bias in varying infant exposure to SARS-CoV-2, only those whose parents had a confirmed SARS-CoV-2 infection from the infant’s date of birth up to 6 months of age were included (Figure 1). By selecting only infants with definite infant exposure to the virus due to the close contact between parents and newborn infants, we limited the possibility of the healthy vaccinee bias and overestimation of estimated vaccine effectiveness for infants, an important limitation of observational studies investigating maternal vaccine effectiveness.^19,20^ This selection was done independent of information regarding maternal vaccination status.

Data were collected from official databases maintained by the Singapore Ministry of Health (MOH). We obtained deidentified data of infants born during the study period from the national Registry of Births and Deaths. The vaccination status of the infants’ biological mother at time of delivery was obtained from the National Immunization Registry, which is maintained by the MOH. SARS-CoV-2 testing with polymerase chain reaction (PCR) or antigen rapid test (ART) was conducted on individuals who presented to a health care facility with acute respiratory symptoms (cough, runny nose, sore throat, and/or fever), and all positive tests are required by law to notify the MOH. Data on the biological mothers’ age, gender, ethnicity, and education level were extracted from official COVID-19 infection and vaccination databases maintained by MOH. Parent and infant data were linked via parent identification numbers reported on the infant’s birth certificate. The study was conducted under the Infectious Diseases Act, Singapore, for policy decision-making; hence, a separate ethics review by an institutional review board was not required. This report follows the Strengthening the Reporting of Observational Studies in Epidemiology (STROBE) reporting guideline.

### Maternal Vaccination Status

National guidelines recommend that persons aged 12 years and older, including pregnant people, receive a third vaccine dose (booster) 5 months after completion of the primary series. Therefore, vaccination coverage in pregnant people was categorized into 3 groups in the study: unvaccinated (never received vaccination since the start of the pandemic), vaccinated prior to pregnancy (completed second dose and/or received a third dose [booster] prior to pregnancy), and vaccinated during pregnancy (completed second dose and/or received a third dose [booster] during pregnancy.

Maternal vaccination status was defined based on the number of doses taken as of 14 days prior to delivery. We chose an interval of 14 days or more to allow adequate time for antibody levels to increase after immunization and to facilitate comparison with the results of previously reported studies of vaccine efficacy.^4,5,20,21^ Infants were categorized by whether they were born to mothers who were unvaccinated, vaccinated prior to pregnancy, or vaccinated during pregnancy.

### Infection Status

The outcome of this study was laboratory-confirmed infant SARS-COV-2 infection of any severity. In accordance with national guidelines, all infants presenting to a clinic or hospital with clinical suspicion for SARS-CoV-2 infection during this period required PCR testing administered by a health care clinician. They were considered to be infected if they had a positive PCR test at any time before turning 7 months of age. Singapore implemented a strict contact tracing, test, and isolate policy as part of public health measures to control the pandemic since the beginning. Testing close contacts, such as household members, was recommended and ART kits were freely distributed nationwide for all households.

### Statistical Analysis

We identified maternal ethnicity and education level as a proxy for infant race and ethnicity and socioeconomic status and extracted these data as important covariates that influence clinical outcomes. The ethnicity categories of Chinese, Indian, Malay, and others were used in accordance with official categories registered in the Singapore National Registration Identification Card system. Other ethnicities may include Eurasian, White, or Arab, Eurasian, White, or others. Gestation week at time of birth was extracted to categorize infants as preterm (32-36 weeks) or normal term (≥37 weeks), as prematurity is associated with substantial comorbidities. Differences in maternal age, ethnicity, parity (1, 2, 3, or 4 or more), education level (up to 10 years of formal education, more than 10 years of formal education without a university degree, university graduate and above) and gestation week at time of birth among infants who were born to mothers who were unvaccinated, vaccinated prior to pregnancy, or vaccinated during pregnancy were analyzed using the χ^2^ test (Table 1). Testing was 2-sided; the significance threshold was *P* < .05.

**Table 1.** Baseline Maternal Sociodemographic and Infant Characteristics

Characteristic	Unadjusted					Adjusted^a^				
	Participants, No. (%)					Person-days, No. (%)				
	Group 1 (n = 172)^b^	Group 2 (n = 578)^c^	Group 3 (n = 6542)^d^	*P* value for group 1 vs group 2	*P* value for group 1 vs group 3	Group 1 (n = 293 299)^b^	Group 2 (n = 570 349)^c^	Group 3 (n = 997 509)^d^	*P* value for group 1 vs group 2	*P* value for group 1 vs group 3
Maternal age, y										
<25	6 (3.5)	18 (3.1)	171 (2.6)	.02	.18	9487 (3.2)	24 382 (4.3)	24 848 (2.5)	<.001	<.001
25-29	26 (15.1)	120 (20.8)	1137 (17.4)			35 251 (12.0)	125 995 (22.1)	170 989 (17.1)		
30-34	69 (40.1)	271 (46.9)	2965 (45.3)			130 532 (44.5)	268 718 (47.1)	448 274 (44.9)		
35-39	53 (30.8)	138 (23.9)	1842 (28.2)			90 511 (30.9)	121 580 (21.3)	287 080 (28.8)		
≥40	18 (10.5)	31 (5.4)	427 (6.5)			27 518 (9.4)	29 674 (5.2)	66 319 (6.6)		
Maternal ethnicity^e^										
Chinese	98 (57.0)	311 (53.8)	4113 (62.9)	<.001	<.001	183 125 (62.4)	334 681 (58.7)	627 840 (62.9)	<.001	<.001
Indian	19 (11.0)	53 (9.2)	455 (7.0)			20 671 (7.0)	34 262 (6.0)	75 610 (7.6)		
Malay	38 (22.1)	197 (34.1)	1772 (27.1)			63 487 (21.6)	188 284 (33)	263 229 (26.4)		
Other	17 (9.9)	17 (2.9)	202 (3.1)			26 016 (8.9)	13 123 (2.3)	30 829 (3.1)		
Maternal education level										
≤10 y	33 (19.2)	111 (19.2)	894 (13.7)	.67	.11	45 068 (15.4)	75 728 (13.3)	134 919 (13.5)	<.001	<.001
>10 y	42 (24.4)	160 (27.7)	1789 (27.3)			78 309 (26.7)	183 308 (32.1)	272 759 (27.3)		
Completed university	97 (56.4)	307 (53.1)	3859 (59.0)			169 922 (57.9)	311 314 (54.6)	589 832 (59.1)		
Maternal parity										
1	80 (46.5)	306 (52.9)	3196 (48.9)	.22	.89	136 122 (46.4)	305 594 (53.6)	470 412 (47.2)	<.001	<.001
2	59 (34.3)	152 (26.3)	2227 (34.0)			108 894 (37.1)	158 937 (27.9)	349 998 (35.1)		
3	23 (13.4)	88 (15.2)	772 (11.8)			35 389 (12.1)	79 886 (14.0)	121 108 (12.1)		
≥4	10 (5.8)	32 (5.5)	347 (5.3)			12 894 (4.4)	25 933 (4.5)	55 991 (5.6)		
Infant gestation, wk										
32-36	12 (7.0)	57 (9.9)	414 (6.3)	.25	.73	21 169 (7.2)	64 124 (11.2)	63 651 (6.4)	<.001	<.001
≥37	160 (93.0)	521 (90.1)	6128 (93.7)			272 130 (92.8)	506 225 (88.8)	933 858 (93.6)		

^a^ Inverse probability weighted person-time are adjusted for age, parity, ethnicity, educational level, gestation age, and calendar week.

^b^ Group 1 included infants born to unvaccinated mothers.

^c^ Group 2 included infants born to mothers who completed 2 to 3 doses but none of which was completed during pregnancy.

^d^ Group 3 included infants born to mothers who completed 2 to 3 doses of which at least the last dose was completed during pregnancy.

^e^ Ethnicity was derived from the Singapore National Registration Identification Card system. Other ethnicities may include Arab, Eurasian, White, or others.

To adjust for confounding by demographic and socioeconomic factors and stage of the pandemic, inverse probability weighting was performed. A logistic regression was used to estimate the probability of infants being born to mothers who received a vaccine dose during pregnancy using maternal age, maternal ethnicity, maternal education level, calendar week, and whether mothers had a SARS-CoV-2 infection during pregnancy as covariates. Inverse probability weights were computed as 1 divided by the probability predicted from the logistic regression for infants born to mothers who received a vaccine dose during pregnancy, and 1 divided by 1 minus the probability predicted from the logistic regression for infants born to mothers who did not receive a vaccine dose during pregnancy.

Inverse probability-weighted Cox regressions were used to estimate the hazard ratios of medically attended SARS-CoV-2 infections per person-day, with infants born to mothers who were unvaccinated serving as the reference group, and maternal age, maternal ethnicity, maternal parity, maternal education level, and gestation week as covariates.

Using previously published methods, we estimated vaccine effectiveness (VE) with the equation: 100% × (1−adjusted hazard ratio of SARS-CoV-2 infection).^4,20,22^ Point estimates of VE were reported with 95% CIs.

We conducted 2 secondary analyses. First, we conducted a subgroup analysis of estimated VE by further stratifying infants born to mothers who had been vaccinated prior to pregnancy and during pregnancy to 2 groups: those born to mothers who had received only 2 doses and those born to mothers who received a third dose (booster). Those born to mothers who received a third dose (booster) during pregnancy were also analyzed according to the trimester during which mothers received the dose. Second, we aimed to investigate estimated VE against the Omicron XBB variant (October 1, 2022, to March 31, 2023). Data analysis was performed with Stata version 17.0 (StataCorp) from April to July 2023.

## Results

Among 7292 infants included in this study (Figure 1), 4522 (62.0%) had mothers who were Chinese, 527 (7.2%) had mothers who were Indian, 2007 (27.5%) had mothers who were Malay, and 236 (3.2%) had mothers who were other ethnicity; and 6809 (93.4%) were born at full term. There were 7120 infants (97.6%) born to mothers who had been fully vaccinated or boosted as of 14 days prior to delivery, 2881 infants (39.5%) born to mothers who received their second dose during pregnancy, and 3661 (50.2%) born to mothers who received a third dose (booster) during pregnancy. Table 1 reports the maternal sociodemographic factors of infants included in the analysis. Mothers who were unvaccinated as of 14 days prior to delivery were more likely to be older (41.3% were aged 35 years and older) and of other ethnicity (9.9%) (Table 1). These differences persisted after adjusting for inverse probability weights. Maternal sociodemographic factors and infant characteristics were similar between infants included and excluded in the study (eTable in Supplement 1).

**Figure 2.**
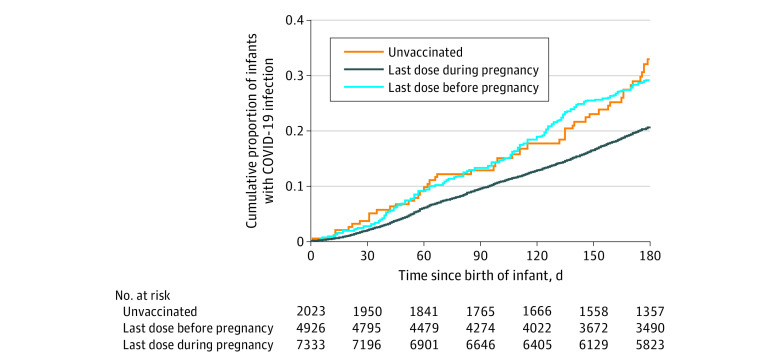
Cumulative Proportion of Infants With SARS-CoV-2 Infection by Maternal Vaccination Status

A total of 1272 infants (17.4%) born to parents who were infected with SARS-CoV-2 post partum also became infected. The crude incidence rate was 174.3 per 100 000 person-days among infants born to unvaccinated mothers, 122.2 per 100 000 person-days among infants born to mothers vaccinated before pregnancy, and 128.5 per 100 000 person-days among infants born to mothers vaccinated during pregnancy. The corresponding estimated VE was 15.4% (95% CI, 17.6% to 39.1%) among infants born to mothers vaccinated before pregnancy, and 41.5% (95% CI, 22.8% to 55.7%) among infants born to mothers vaccinated during pregnancy (Table 2, Figure 2). Among infants born to mothers who were vaccinated during pregnancy, estimated VE was 44.4% (95% CI, 26.2% to 58.1%) if mothers received a third dose (booster), compared with 37.6% (95% CI, 17.2% to 53.1%) if mothers received their second dose (Table 2). There was no significant difference in estimated VE by the trimester during which their mothers received a third dose (booster): 45.5% (95% CI, 22.4% to 61.7%) during the first trimester, 43.8% (95% CI, 24.7% to 58.0%) during the second trimester, and 45.0% (95% CI, 25.8% to 59.2%) during the third trimester. Infants born to mothers who completed 2 doses (estimated VE, 18.3% [95% CI, −14.6% to 41.8%]) or 3 doses (estimated VE, −0.4% [95% CI, −67.5% to 39.8%) prior to their pregnancy did not have a lower risk for SARS-CoV-2 infection.

**Table 2.** Estimated Effectiveness of Messenger RNA Vaccines Against SARS-CoV-2 Infection Stratified by Whether a Dose Was Received During Pregnancy^a^

Group	Person-days at risk	SARS-CoV-2 infection within 6 mo from birth, No.	Crude incidence rate per 100 000 person-days	Estimated vaccine effectiveness, % (95% CI)
Unvaccinated	25 243	44	174.3	[Reference]
Vaccinated before pregnancy	55 654	68	122.2	15.4 (−17.6 to 39.1)
Completed first and second dose only	51 199	67	130.9	18.3 (−14.6 to 41.8)
Completed first, second, and third doses	4455	1	22.4	NA
Vaccinated during pregnancy	903 056	1160	128.5	41.5 (22.8 to 55.7)
Completed first and second dose only	464 383	630	135.7	37.6 (17.2 to 53.1)
Completed first, second, and third doses	438 673	530	120.8	44.4 (26.2 to 58.1)

Abbreviation: NA, not applicable.

^a^ Multivariate Cox regression adjusted for mother’s age, ethnicity, education level, parity, and infants’ gestational age. Estimated vaccine effectiveness = 100% × (1 − adjusted hazard ratio of SARS-CoV-2 infection).

In our Omicron XBB variant–specific VE analysis, there was an association with lower risk for infection observed among infants born to mothers vaccinated with a third dose (booster) during pregnancy. VE against Omicron XBB variant was estimated to be 76.7% (95% CI, 12.8% to 93.8%) (Table 3).

**Table 3.** Estimated Effectiveness of Messenger RNA Vaccines During Wide Circulation of SARS-CoV-2 XBB Variant (October 2022 to March 2023) Stratified by Whether a Dose Was Received During Pregnancy^a^

Group	Person-days at risk	SARS-CoV-2 infection within 6 mo from birth, No.	Crude incidence rate per 100 000 person-days	Vaccine effectiveness, % (95% CI)
Unvaccinated	2275	6	263.7	[Reference]
Vaccinated before pregnancy	17 211	36	209.2	30.8 (−162.6 to 81.8)
Completed first and second dose only	12 544	27	215.2	60.7 (−56.6 to 90.1)
Completed first, second, and third doses	4667	9	192.8	−12.7 (−339.1 to 71.1)
Vaccinated during pregnancy	159 493	245	153.6	74.8 (5.7 to 93.2)
Completed first and second dose only	40 390	65	160.9	73 (−30.6 to 94.4)
Completed first, second, and third doses	119 103	180	151.1	76.7 (12.8 to 93.8)

^a^ Multivariate Cox regression adjusted for mother’s age, ethnicity, education level, parity, and infants’ gestational age. Estimated vaccine effectiveness = 100% × (1 − adjusted hazard ratio of SARS-CoV-2 infection).

## Discussion

In our cohort study of live births in Singapore between January 1, 2022, and September 30, 2023, maternal mRNA SARS-Cov-2 vaccination was associated with a lower risk for SARS-CoV-2 infection, including against Omicron XBB, in infants up to 6 months of age. Overall estimated VE was 41.5% (95% CI, 22.8%-55.7%) if the second dose or third dose (booster) was given during pregnancy. However, we found that vaccination including receipt of third dose (booster) before pregnancy was not associated with a lower risk of infection with Omicron variants in infants. There may be a need for mRNA SARS-CoV-2 vaccination to be recommended for every pregnancy similar to maternal influenza and pertussis vaccination in order to maintain protection in newborns.

In a national cohort study from Norway, estimated VE was 33% during an Omicron-dominated period.^20^ In contrast, a study in Northern California reported a 13% lower risk of infection during Omicron from maternal vaccination but this was not statistically significant.^23^ A Canadian study reported an estimated VE of 73% against Omicron infection.^13^ However, this higher estimate was based on infections from May 2021 and did not cover the Omicron XBB wave which has been shown to have mutations more capable of evading vaccine-induced immunity compared with previous Omicron variants.^24^ Our study, which was designed to reduce bias from differences in SARS-CoV-2 exposure by only including infants of parents who had a confirmed SARS-CoV-2 infection during the first 6 months after delivery, adds to the evidence on the association of maternal vaccination during pregnancy with a lower risk of infants acquiring SARS-CoV-2 infection. In addition, we identified in our Omicron XBB variant–specific analysis the need for a third dose (booster) during pregnancy for the lower risk of infection to be maintained. This requires further study as we used a predetermined Omicron XBB community circulation period (October 2022 to April 2023) rather than whole-genome sequencing of confirmed cases in the analysis.^25^

Previous studies primarily looked at VE from receipt of vaccination during pregnancy. Pregnant people who received vaccination prior to pregnancy was an exclusion criterion in the Canadian study. Therefore, data comparing protection in infants derived from vaccination during pregnancy vs prior to pregnancy is scarce. A recent study from Israel reported that a third dose (booster) during pregnancy was needed for 46% protection against SARS-CoV-2 hospitalization in infants for up to 4 months. Interestingly, they found that infants of mothers who had 2 doses prior to pregnancy were not protected against SARS-CoV-2 hospitalizations (VE, −16% [95% CI, −56% to −14%]).^14^ Our study corroborated this finding as infants of mothers who received vaccination prior to pregnancy remained at a higher risk of infection (VE, 15.4% [95% CI, −17.6% to 39.1%) compared with infants of mothers who received vaccination during pregnancy (VE, 41.5% [95% CI, 22.8% to 55.7%]). Infants derive direct protection from maternal vaccination through acquisition of antibodies via the transfer of antibodies across the placenta during pregnancy and consumption of milk via breast feeding. The amount of anti-SARS-CoV-2 antibodies transferred from mother to umbilical cord blood at birth is dependent on the level of antibodies in the mother and this wanes with time.^26,27^ High levels of neutralizing antibodies are required for protection against infection by Omicron variants.^28,29^ Hence, mothers who did not receive vaccination during pregnancy are likely to have a lower level of antibodies compared with those who received vaccination during pregnancy. This may be 1 of the reasons for the observed lack of lower risk for Omicron variant infection in our study and the Israeli study.^14^ Worryingly, we found that receipt of the third dose (booster) prior to pregnancy did not overcome this lack of lower risk among infants for SARS-CoV-2 infection (VE, −0.4% [95% CI, −67.5% to 39.8%]). This requires confirmation from other studies as there was only 1 SARS-CoV-2 infection among infants whose mother received a third dose (booster) prior to pregnancy in the cohort. If confirmed, there may be a need for maternal SARS-CoV-2 vaccination to be recommended for every pregnancy similar to current maternal vaccination recommendations for influenza and pertussis.

The strength of our cohort study was that it used nationwide data on vaccination rates with legally mandated records of SARS-CoV-2 infection rates to assess the entire nation’s resident infant population. There was consistency and reproducibility in case definition as it was nationally required that infants infected with SARS-CoV-2 had to be reviewed by a physician and notified. An added strength is that our methods only included those whose parent or parents in the same household had a confirmed SARS-CoV-2 infection, which minimized the confounding effect of variations in infant SARS-CoV-2 exposure. The close contact between parents and newborn infant ensured that biases resulting from changes in community transmission and self-isolation behavior were mitigated. As an added precaution, we included a calendar-time variable as a surrogate for evolving community infection rates and social distancing measures in our multivariate regression model. To date, most studies observed infant SARS-CoV-2 infections before the rise of the Omicron XBB variant in late 2022, whereas our study extended to March 2023 and hence was able to study estimated VE and infant Omicron XBB variant infections.

### Limitations

Our study has several limitations. There could be an added protective effect of breastfeeding which would increase the transplacental transfer of SARS-CoV-2 antibodies through breastmilk.^30,31^ However, this information was not available in our cohort. Infant determinants of heath such as prematurity and the Apgar score, which is a summary measure of infant health at delivery, were not available for analysis. However, we mitigated this by including only infants born after 32 weeks of gestation. We omitted studying the effect of maternal vaccination on hospitalization for SARS-CoV-2 as admission guidelines in Singapore were dynamic during this observation period and may not be an accurate reflection of disease severity. We could not account for differences in individual infection control measures implemented by parents with SARS-CoV-2 infection to protect their infants. However, the close bond between parents, especially mothers, and newborns would likely limit the effectiveness of wearing masks. Furthermore, SARS-CoV-2 infection is known to transmit prior to onset of symptoms which would also limit the effect of any infection control measures taken after confirmation.

## Conclusions

This cohort study found that maternal mRNA SARS-CoV-2 vaccination administered during pregnancy had an estimated VE of 41.5% in reducing the risk of Omicron SARS-CoV-2 infection in infants up to 6 months of age. A third dose (booster) during pregnancy may be needed to maintain protection specifically against the Omicron XBB variant. Importantly, we also found that infants of mothers who received 2 or even 3 doses of vaccination prior to pregnancy remained susceptible to Omicron SARS-CoV-2 infections. Therefore, pregnant people should not depend on vaccines received prior to their pregnancy but are strongly encouraged to consider completing any remaining recommended vaccination doses, including booster doses during pregnancy. Aside from protecting mothers from infection, these booster doses could ensure continued protection of their newborn infants after delivery. If corroborated by other studies, our findings that the lower risk was only maintained in infants when their mothers were vaccinated during pregnancy may suggest a need for maternal SARS-CoV-2 vaccination at each pregnancy similar to current recommendations for maternal influenza and pertussis vaccination.

## Notes

Supplement 1. **eTable.** Comparison of Baseline Maternal Sociodemographic and Infant Characteristics Between Infants Who Were Included and Excluded Based on Parental Infection Status

Supplement 2.
Data Sharing Statement

## References

[1] Cui X, Zhao Z, Zhang T, . A systematic review and meta-analysis of children with coronavirus disease 2019 (COVID-19). J Med Virol. 2021;93(2):1057-1069. doi:10.1002/jmv.26398 32761898 PMC7436402

[2] Hobbs CV, Woodworth K, Young CC, ; for the Overcoming COVID-19 Investigators. Frequency, characteristics and complications of COVID-19 in hospitalized infants. Pediatr Infect Dis J. 2022;41(3):e81-e86. doi:10.1097/INF.0000000000003435 34955519 PMC8828316

[3] Marks KJ, Whitaker M, Agathis NT, ; COVID-NET Surveillance Team. Hospitalization of infants and children aged 0-4 years with laboratory-confirmed COVID-19 - COVID-NET, 14 states, March 2020-February 2022. MMWR Morb Mortal Wkly Rep. 2022;71(11):429-436. doi:10.15585/mmwr.mm7111e2 35298458 PMC8942304

[4] Tan SHX, Cook AR, Heng D, Ong B, Lye DC, Tan KB. . Effectiveness of BNT162b2 vaccine against Omicron in children 5 to 11 years of age. N Engl J Med. 2022;387(6):525-532. doi:10.1056/NEJMoa2203209 35857701 PMC9342421

[5] Walter EB, Talaat KR, Sabharwal C, ; C4591007 Clinical Trial Group. Evaluation of the BNT162b2 Covid-19 vaccine in children 5 to 11 years of age. N Engl J Med. 2022;386(1):35-46. doi:10.1056/NEJMoa2116298 34752019 PMC8609605

[6] Yung CF, Pang D, Kam KQ, . BNT162b2 vaccine protection against omicron and effect of previous infection variant and vaccination sequence among children and adolescents in Singapore: a population-based cohort study. Lancet Child Adolesc Health. 2023;7(7):463-470. doi:10.1016/S2352-4642(23)00101-3 37201540 PMC10185330

[7] Dagan N, Barda N, Biron-Shental T, . Effectiveness of the BNT162b2 mRNA COVID-19 vaccine in pregnancy. Nat Med. 2021;27(10):1693-1695. doi:10.1038/s41591-021-01490-8 34493859

[8] Butt AA, Chemaitelly H, Al Khal A, . SARS-CoV-2 vaccine effectiveness in preventing confirmed infection in pregnant women. J Clin Invest. 2021;131(23):e153662. doi:10.1172/JCI153662 34618693 PMC8631593

[9] Regan AK, Munoz FM. Efficacy and safety of influenza vaccination during pregnancy: realizing the potential of maternal influenza immunization. Expert Rev Vaccines. 2021;20(6):649-660. doi:10.1080/14760584.2021.1915138 33832397

[10] Campbell H, Gupta S, Dolan GP, . Review of vaccination in pregnancy to prevent pertussis in early infancy. J Med Microbiol. 2018;67(10):1426-1456. doi:10.1099/jmm.0.000829 30222536

[11] Shook LL, Atyeo CG, Yonker LM, . Durability of anti-spike antibodies in infants after maternal COVID-19 vaccination or natural infection. JAMA. 2022;327(11):1087-1089. doi:10.1001/jama.2022.1206 35129576 PMC8822441

[12] Mithal LB, Otero S, Shanes ED, Goldstein JA, Miller ES. Cord blood antibodies following maternal coronavirus disease 2019 vaccination during pregnancy. Am J Obstet Gynecol. 2021;225(2):192-194. doi:10.1016/j.ajog.2021.03.035 33812808 PMC8012273

[13] Jorgensen SCJ, Hernandez A, Fell DB, ; Canadian Immunization Research Network (CIRN) Provincial Collaborative Network (PCN) Investigators. Maternal mRNA covid-19 vaccination during pregnancy and delta or omicron infection or hospital admission in infants: test negative design study. BMJ. 2023;380:e074035. doi:10.1136/bmj-2022-074035 36754426 PMC9903336

[14] Lipschuetz M, Guedalia J, Cohen SM, . Maternal third dose of BNT162b2 mRNA vaccine and risk of infant COVID-19 hospitalization. Nat Med. 2023;29(5):1155-1163. doi:10.1038/s41591-023-02270-2 36959421

[15] Rahmati M, Yon DK, Lee SW, . Effects of COVID-19 vaccination during pregnancy on SARS-CoV-2 infection and maternal and neonatal outcomes: a systematic review and meta-analysis. Rev Med Virol. 2023;33(3):e2434. doi:10.1002/rmv.2434 36896895

[16] Cui Y, Binger K, Palatnik A. Attitudes and beliefs associated with COVID-19 vaccination during pregnancy. JAMA Netw Open. 2022;5(4):e227430. doi:10.1001/jamanetworkopen.2022.7430 35420664 PMC9011126

[17] Centers for Disease Control and Prevention. Archived cumulative data. percent of pregnant people aged 18-49 years receiving at least one dose of a COVID-19 vaccine during pregnancy overall, by race/ethnicity, and date reported to CDC-Vaccine Safety Datalink*, United States | December 20, 2020 – Jan. Accessed October 2022. https://data.cdc.gov/Vaccinations/Archived-Cumulative-Data-Percent-of-pregnant-peopl/4ht3-nbmd

[18] MOH. Update on COVID-19 situation and measures to protect healthcare capacity. Accessed July 10, 2023. https://www.moh.gov.sg/news-highlights/details/update-on-covid-19-situation-and-measures-to-protect-healthcare-capacity

[19] Remschmidt C, Wichmann O, Harder T. Frequency and impact of confounding by indication and healthy vaccinee bias in observational studies assessing influenza vaccine effectiveness: a systematic review. BMC Infect Dis. 2015;15(1):429. doi:10.1186/s12879-015-1154-y 26474974 PMC4609091

[20] Carlsen EØ, Magnus MC, Oakley L, . Association of COVID-19 vaccination during pregnancy with incidence of SARS-CoV-2 infection in infants. JAMA Intern Med. 2022;182(8):825-831. doi:10.1001/jamainternmed.2022.2442 35648413 PMC9161123

[21] Frenck RW Jr, Klein NP, Kitchin N, ; C4591001 Clinical Trial Group. Safety, immunogenicity, and efficacy of the BNT162b2 Covid-19 vaccine in adolescents. N Engl J Med. 2021;385(3):239-250. doi:10.1056/NEJMoa2107456 34043894 PMC8174030

[22] Bar-On YM, Goldberg Y, Mandel M, . Protection of BNT162b2 vaccine booster against Covid-19 in Israel. N Engl J Med. 2021;385(15):1393-1400. doi:10.1056/NEJMoa2114255 34525275 PMC8461568

[23] Zerbo O, Ray GT, Fireman B, . Maternal SARS-CoV-2 vaccination and infant protection against SARS-CoV-2 during the first six months of life. Nat Commun. 2023;14(1):894. doi:10.1038/s41467-023-36547-4 36854660 PMC9974935

[24] Cao Y, Jian F, Wang J, . Imprinted SARS-CoV-2 humoral immunity induces convergent Omicron RBD evolution. Nature. 2023;614(7948):521-529. 36535326 10.1038/s41586-022-05644-7 PMC9931576

[25] Goh AXC, Chae S-R, Chiew CJ, . Characteristics of the omicron XBB subvariant wave in Singapore. Lancet. 2023;401(10384):1261-1262. doi:10.1016/S0140-6736(23)00390-2 37061259

[26] Rottenstreich A, Zarbiv G, Oiknine-Djian E, . Kinetics of maternally derived anti-severe acute respiratory syndrome coronavirus 2 (SARS-CoV-2) antibodies in infants in relation to the timing of antenatal vaccination. Clin Infect Dis. 2023;76(3):e274-e279. doi:10.1093/cid/ciac480 35717644 PMC9214162

[27] Atyeo CG, Shook LL, Brigida S, . Maternal immune response and placental antibody transfer after COVID-19 vaccination across trimester and platforms. Nat Commun. 2022;13(1):3571. doi:10.1038/s41467-022-31169-8 35764643 PMC9239994

[28] Khoury DS, Cromer D, Reynaldi A, . Neutralizing antibody levels are highly predictive of immune protection from symptomatic SARS-CoV-2 infection. Nat Med. 2021;27(7):1205-1211. doi:10.1038/s41591-021-01377-8 34002089

[29] Yung CF, Bert NL, Kam KQ, . BNT162b2 vaccine induced variant-specific immunity, safety and risk of Omicron breakthrough infection in children aged 5 to 11 years: a cohort study. Research Square. Accessed June 26, 2023. https://www.researchsquare.com/article/rs-2928224/v1 10.1038/s41598-023-44565-x PMC10575958 37833554

[30] Perl SH, Uzan-Yulzari A, Klainer H, . SARS-CoV-2-specific antibodies in breast milk after COVID-19 vaccination of breastfeeding women. JAMA. 2021;325(19):2013-2014. doi:10.1001/jama.2021.5782 33843975 PMC8042567

[31] Gray KJ, Bordt EA, Atyeo C, . Coronavirus disease 2019 vaccine response in pregnant and lactating women: a cohort study. Am J Obstet Gynecol. 2021;225(3):303.e1-303.e17. doi:10.1016/j.ajog.2021.03.023 33775692 PMC7997025

