# Supplementary material for: mRNA SARS-CoV-2 Vaccination Before vs During Pregnancy and Omicron Infection Among Infants
Source: JAMA Netw Open. 2023 Nov 10;6(11):e2342475. doi: 10.1001/jamanetworkopen.2023.42475 (PMC10638647; doi:10.1001/jamanetworkopen.2023.42475)
Supplement: Supplement 1. — eTable. Comparison of Baseline Maternal Sociodemographic and Infant Characteristics Between Infants Who Were Included and Excluded Based on Parental Infection Status [file jamanetwopen-e2342475-s001.pdf]

Supplemental Online Content

Goh O, Pang D, Tan J, et al. mRNA SARS-CoV-2 vaccination before vs during pregnancy and Omicron infection among infants. *JAMA Netw Open.* 2023;6(11):e2342475. doi:10.1001/jamanetworkopen.2023.42475

eTable. Comparison of Baseline Maternal Sociodemographic and Infant Characteristics Between Infants Who Were Included and Excluded Based on Parental Infection Status

This supplemental material has been provided by the authors to give readers additional information about their work.

© 2023 Goh O et al. *JAMA Network Open.*

eTable. Comparison of Baseline Maternal Sociodemographic and Infant Characteristics Between Infants Who Were Included and Excluded Based on Parental Infection Status

		**Parents infected after birth n=7,292**	**Parents not infected after birth n=13,611**	**P value***
Maternal age (%)	<25	2.7	3.4	0.011
	25-29	17.6	18.4	
	30-34	45.3	44.8	
	35-39	27.9	26.7	
	>=40	6.5	6.6	
Ethnicity (%)	Chinese	62.0	61.2	<0.001
	Indian	7.2	8.4	
	Malay	27.5	25.7	
	Others	4.8	3.2	
Education level (%)	Did not complete university	41.5	40.5	<0.001
	Completed university	58.5	59.4	
Maternal parity (%)	1	49.1	50.7	0.002
	2	33.4	31.1	
	3	12.1	12.1	
	>=4	5.3	6.1	
Gestation week (%)	32-36 weeks	6.6	7.5	0.022
	>=37 weeks	93.4	92.5	

* Differences in proportion were analysed using chi^2 testing

© 2023 Goh O et al. *JAMA Network Open.*
