# Supplementary material for: mRNA SARS-CoV-2 Vaccination Before vs During Pregnancy and Omicron Infection Among Infants
Source: JAMA Netw Open. 2023 Nov 10;6(11):e2342475. doi: 10.1001/jamanetworkopen.2023.42475 (PMC10638647; doi:10.1001/jamanetworkopen.2023.42475)
Supplement: Supplement 2. — Data Sharing Statement [file jamanetwopen-e2342475-s002.pdf]

Data Sharing Statement

Goh. mRNA SARS-CoV-2 Vaccination Before vs During Pregnancy and Omicron Infection Among Infants. *JAMA Netw Open*. Published November 09, 2023. doi:10.1001/jamanetworkopen.2023.42475

Data
Data available: No

Additional Information
Explanation for why data not available: Requests for available data may be directed to the corresponding author
